# Contamination of Kazakhstan cheeses originating from *Escherichia coli* and its resistance to antimicrobial drugs

**DOI:** 10.14202/vetworld.2024.361-370

**Published:** 2024-02-15

**Authors:** Anar Kuzeubayeva, Altay Ussenbayev, Ali Aydin, Zhannara Akanova, Raushan Rychshanova, Elmira Abdullina, Dinara Seitkamzina, Laura Sakharia, Saidulla Ruzmatov

**Affiliations:** 1 Department of Veterinary Medicine and Livestock Technology, Seifullin Kazakh Agrotechnical Research University, Astana, 010000, Kazakhstan; 2 Department of Food Hygiene and Technology, Faculty of Veterinary Medicine, Istanbul University - Cerrahpaşa, Istanbul, 34320, Turkey; 3 Scientific Innovation Center, Research Institute of Applied Biotechnology, A. Baitursynov Kostanay Regional University, Kostanay, 110000, Kazakhstan; 4 Department of Veterinary Sanitation, Shakarim University of Semey, Semey, 071412, Kazakhstan

**Keywords:** antibiotic resistance, *Escherichia coli* O157:H7, Kazakhstan cheese, microbial contamination

## Abstract

**Background and Aim:**

*Escherichia coli*, a commensal intestine bacterium of vertebrates, is widely distributed in the environment and indicates the microbiological quality of food products in relation to coliforms. In addition, virulent strains, particularly *E. coli* O157:H7, cause outbreaks of toxic infections caused by consuming dairy products. Because food safety studies regarding *E. coli* have not been conducted in Central Asia, this research aimed to study the characteristics of contamination, microbiological and genotypic properties, and resistance to antimicrobial agents of *E. coli* strains that contaminate various types of commercialized cheeses originating from Kazakhstan.

**Materials and Methods:**

In retail outlets, 207 samples of three types of cheese produced by 22 industrial and eight small enterprises in the central, eastern, southern, and northern regions of Kazakhstan were selected in 2020–2023. *E. coli* contamination was examined using standard microbiological, mass spectrometric, and molecular genetic methods. The discodiffuse European Committee on Antimicrobial Susceptibility Testing method was used to test the resistance of the identified *E. coli* isolates (65/207; 31.4%) to 20 antibacterial drugs. The Shiga toxin-producing *E. coli* (*VT1 and VT2*) and *E. coli* O157:H7 (*eae*) genes were investigated in all *E. coli* isolates using multiplex polymerase chain reaction.

**Results:**

An average of 31.4% samples of commercial Kazakhstani cheeses of various types were found to be contaminated with *E*. *coli* in almost all geographical regions of Kazakhstan, regardless of the productivity of the dairy enterprises. Soft cheeses produced by small farms (80% of samples) packaged at the retail site (100%) were the most contaminated with *E*. *coli*. The microbiological index (colony-forming unit/g) was unsatisfactory and unsuitable in 6.2% of such cheese samples. For the first time in Central Asia, the enteropathogenic strain *E. coli* O157:H7 was detected in 0.5% of cheese samples. *E*. *coli* isolates from cheese samples were resistant to 65% of antibacterial drugs and contained resistance genes to β-lactams, sulfonamides, and quinolones groups. At the same time, 25% of the *E*. *coli* isolates were multi-resistant to three or more antimicrobial agents.

**Conclusion:**

The high level of contamination caused by multi-antibiotic resistant *E. coli* strains, including pathogenic pathogens, poses a risk to public health and highlights the need for further research on the monitoring and control of coliform enteropathogens in food products.

## Introduction

There is an increase in global consumer demand for cheeses, which are considered the most useful source of vital nutrients, such as vitamins, minerals, and proteins, which constitute the main part of healthy foods [1]. Cheese production is one of the main sectors of the dairy industry and cheeses are usually made from raw or pasteurized milk [2, 3]. According to the Bureau of National Statistics of Kazakhstan, in 2022, 38 thousand tons of cheese were produced in the country, the production of which has increased by 50% over the past 5 years due to the expansion of market capacity after the growth trend of urbanization. In general, the consumption of cheese is 2.1 kg/person/year, and it is considered the preferred product in the everyday and festive diet of Kazakhstani consumers (www: statgov.kz).

Although cheeses are considered to be microbiologically safe food products, outbreaks of toxic and infectious diseases associated with the contamination of these dairy products with pathogenic bacteria have been regularly recorded in different regions of the world [4]. In this respect, cheeses made from raw milk are particularly dangerous [5]. This indicates the urgency of the problem of bacterial contamination, given that, as mentioned above, global and domestic demand for these substances continues to grow. It has been shown that pathogenic bacteria in cheeses can persist due to their survival during production. In addition, dangerous microorganisms may enter the cheese at retail outlets where cutting and packaging take place, and sanitary conditions are not sufficient to prevent re-contamination [6, 7].

*Escherichia coli*, a Gram-negative, non-spore-forming bacterium of the *Enterobacteriaceae* family [8], is the most common microorganism transmitted through dairy products. In addition to *Escherichia*, the common genera of this family, which also contaminate cheeses and other products, are *Citrobacter*, *Enterobacter*, *Klebsiella*, etc. [9–11]. It is well known that samples of dairy products contaminated by *E. coli* usually contain a significantly higher number of these bacterial genera and the overall organoleptic characteristics of the products are low. Thus, *E. coli* serves as an indicator for assessing the microbiological quality of dairy products, indicating the presence of other food enteropathogenic pathogens [10, 12].

*E. coli* is an important representative of the normal intestinal microflora of humans and other mammals [13]. However, this species has at least six pathotypes that cause a variety of intestinal and extraintestinal disorders. According to the virulence factor, bacteria of this species can be divided into several groups, including enterotoxigenic *E. coli*, attaching and effacing *E. coli*, enteropathogenic *E. coli* (EPEC), enterohemorrhagic *E. coli*, and Shiga toxin-producing *E. coli* (STEC)/verotoxins-producing *E. coli* [14]. Cheese producers are concerned about *E. coli*, which produces shiga/verotoxins encoded by th *STX* gene [15, 16]. Sporadic infections caused by the *E. coli* O157:H7 strain have become an important cause of endemic disease outbreaks in Europe [17]. *E. coli* pathotypes are recognized as significant foodborne pathogens; therefore, rapid identification of *E. coli* pathotypes is important for food hygiene management and rapid epidemiological actions.

In addition to traditional methods of microbiological cultivation based on morphological and biochemical characteristics, molecular biological methods have become a priority in the study of the *Enterobacteriaceae* family for species identification, epidemiological typing, and determination of a phylogenetic relationship between pathogenic and nonpathogenic bacteria. In addition, due to its practical significance, bacteria of this family can transfer and transmit genes of resistance to antibacterial drugs to other microorganisms in the human and animal gut microbiome [18]. At present, antibiotic resistance is recognized as a significant public health problem and poses a threat to human health [19, 20].

The epidemiological characteristics and antibiotic resistance of microbial contamination of cheeses with *E*. *coli* species in Central Asia have not yet been investigated. Therefore, it is of scientific and practical interest to identify the food safety of domestic producers’ cheeses in relation to pathogenic strains of *E*. *coli* and other *Enterobacteriaceae* family microorganisms.

Therefore, this study aimed to identify the epidemiological features, phenotypic and genotypic properties, and resistance to antimicrobial agents of *E. coli* strains that contaminate various cheeses produced in Kazakhstan.

## Materials and Methods

### Ethical approval

The study goal does not require ethical approval because the study was conducted on cheese samples.

### Study period and location

**Figure-1:**
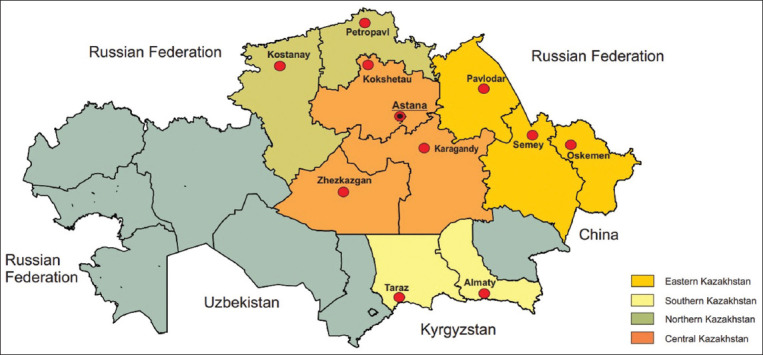
Geographical locations of sampling (red marking shows the area from where the samples were collected).

From September 2020 to April 2023, cheese samples were collected at retail outlets and farmers’ markets in the central, eastern, southern, and northern regions of Kazakhstan (Figure-1 and Table-1).

**Table-1:** Sampling of products under study (by type of cheese).

(a) Distribution of samples by production regions							

Type of cheese	Eastern Kazakhstan, n (%)	Central Kazakhstan, n (%)		Northern Kazakhstan, n (%)	Southern Kazakhstan, n (%)	Total number of samples	
Soft	26 (41)	12 (19)		22 (34)	4 (6)	64	
Semi-hard	47 (46)	29 (28)		23 (23)	3 (3)	102	
Hard	14 (34)	15 (37)		9 (22)	3 (7)	41	
	87 (42)	56 (27)		54 (26)	10 (5)	207	

**(b) According to the method of commercial realization**							

**Type of cheese**	**Individual vacuum packaging, n (%)**						**Cutting, package, n (%)**

Soft	58 (90)						6 (10)
Semi-hard	76 (75)						26 (25)
Hard	27 (66)						14 (34)
Total number of samples	161 (78)						46 (22)

Microbiological and molecular genetic studies were conducted at the Kazakh-Chinese Biosafety Laboratory of the S. Seifullin Kazakh Agrotechnical Research University, the Laboratory of Molecular Diagnostics and Food Safety of Serrahpasha Istanbul University, the Research Institute of Applied Biotechnology of the A. Baitursynov Kostanay Regional University, and the National Center of Biotechnology of Kazakhstan.

### Sampling

In the studied regions, 207 samples of three types of cheese (hard, semi-hard, and soft) from 15 brands, 22 industrial enterprises, and eight small farm producers were collected by random selection. At the same time, 78% and 22% of the samples were sold in factory individual vacuum packaging and cut and placed in plastic bags after purchase, respectively (Table-1).

Samples collected under aseptic conditions were delivered to the laboratory in an ice box and microbiological examination was carried out on the day of admission. The remaining volume of samples was stored at −20°C for further research.

### Microbiological studies

Primary microbiological studies of cheese samples for the presence of *E*. *coli* were performed using Compact Dry EC commercial plates (R-Biopharm AG, Japan) according to the instructions.

Mass spectrometric analysis of isolated bacterial cultures from 93 samples was performed in a linear mode on matrix-assisted laser desorption ionization (MALDI) Biotyper 4.0, Bruker Daltonics (Germany) for species identification. For this purpose, cultures of microorganisms (single colonies) were applied to the cells of a 96-well MSP chip (MSP 96 target polished steel BC, microScoutTarget). Next, 1 mL of matrix solution (saturated solution of a-Cyano-4-hydroxycoric acid with 50% acetonitrile and 2.5% trifluoroacetic acid) was applied and dried at 20–25^0^C. The chip deposited with the samples was placed in a MALDI–time of flight microflex LT mass spectrometer (Bruker Daltonics). After positioning the chip in the ionization chamber and reaching the required values of the operating parameters of the device, calibration was performed using the applied calibration standard. Then, the spectra were collected in automatic mode. To obtain a single mass spectrum, 40 laser pulses (60 Hz frequency) were used. The mass/charge range analyzed was 2000–20,000 Da. Mass spectra were recorded, processed, and analyzed using flexControl software, MALDI Biotyper version 3.0, and MALDI Biotyper RTC (Bruker Daltonik).

A microbiological study of 65 cheese samples, in which the presence of *E*. *coli* cultures was confirmed, was carried out by sowing on selective media with re-sowing on differential diagnostic media; T.B.X. agar (Merck KGaA, Darmstadt, Germany), Chromagar™ E. coli (CHROMagar™, Paris, France), McConkey (Merck KGaA), Eosin Methylene Blue agar (Merck KGaA), Nutrient agar (Azimut, Saint Petersburg, Russia), and biochemical testing in accordance with conventional domestic and international standards (GOST 30726-2001, ISO 16649-2:2001) [21, 22]. Isolated *E. coli* colonies were frozen in nutrient broth (15% glycerin) and stored at −80°C for further use.

### Identification of the pathogenic *E. coli* strains

Isolates from 65 cheese samples were tested for the presence of STEC and *E. coli* O157:H7 strains by determining *STX* (*VT1 and VT2*) and *eae* genes in DNA using known primers (Table-2) in multiplex polymerase chain reaction (PCR) according to the methodology described in the Terrestrial Manual of the World Organization for Animal Health [23].

**Table-2:** Primers used to confirm the presence of virulence determinants in the multiplex PCR.

Target gene	Registration number	Sequence of primers	Nucleotide position	Amplicon size (bp)
*VT1*	M19437 F	CGC-TCT-GCA-ATA-GGT-ACT-CC	287–306	256
	M19437 R	CGC-TGT-TGT-ACC-TGG-AAA-GG	522–541	
*VT2*	X07865 F	TCC-ATG-ACA-ACG-GAC-AGC-AG	623–642	185
	X07865 R	GC-TTC-TGC-TGT-GAC-AGT-GAC	788–807	
*eae*	X60439 F	GC-TTA-GTG-CTG-GTT-TAG-GAT-TG	271–293	618
	X60439 R	CCA-GTG-AAC-TAC-CGT-CAA-AG	871–890	

PCR=Polymerase chain reaction

For DNA extraction from *E*. *coli* colonies, the QIAmp DNA Mini Kit (Qiagen, Germany) was used according to the manufacturer’s instructions. DNA concentration was measured using a NanoDrop 1000 spectrophotometer at a wavelength of 260 nm, and a qualitative assessment of DNA was performed by electrophoresis.

The multiplex PCR reaction was performed in a total volume of 48 µL, including 3 mM MgCl_2_, 3 mM buffer KCL, 3 mM dNTP 2 mM, 25 pmol of each primer (forward and reverse primers for VT1, VT2, and *eaeA*), 0.2 Taq polymerase, and 3 mL (40–260 ng/mL) DNA. The amplification reaction was carried out using a DNA thermal cycler (Eppendorf Mastercycler 5330), which included denaturation for 2 min at 94°C; 28 cycles: 94°C–1 min, 62°C–1.5 min, and 72°C–2 min; and final elongation at 72°C–5 min. Amplified samples were electrophoresed in 1.5% agarose gel and stained with ethidium bromide. We used a molecular weight marker with a step length of 100 bp as the size standard.

### Detection of antibiotic resistance

The resistance of the identified *E*. *coli* isolates to antibacterial drugs was tested using the European Committee on Antimicrobial Susceptibility Testing (EUCAST) Disk Diffusion Method for Antimicrobial Suspicion Testing Version 9.0 (January 2021) using Muller–Hinton agar (HiMedia Laboratories, India). The isolates were diluted in normal 0.85% saline solution to a density of 0.5 according to the McFarland turbidity standard. Subculture in a volume of 100 μL was applied to the surface of the agar. The following discs were used for testing (Pasteur Institute of Epidemiology and Microbiology, Russia): beta-lactams (ampicillin [AMP] – 10 µg, amoxicillin [AMX]– 25 µg, cefoperazone [CFP] – 75 µg, cefoxitin – 30 µg, cefpodoxime [CFD] – 10 µg), meropenem [MPN] – 10 µg, aminoglycosides (streptomycin [STR] – 10 µg, kanamycin (KN) – 30 µg, gentamicin [GNT] – 120 µg), amphenicols (levomycetin [LEV] – 30 µg), tetracyclines (tetracycline (TET) – 30 µg, doxycycline [DOX] – 30 µg), fluoroquinolones (enrofloxacin [EFX] – 5 µg, ciprofloxacin (CFX) – 5 µg, norfloxacin [NFX]– 10 µg, ofloxacin [OFX] – 5 µg), quinolones (nalidixic acid [NA]– 30 µg), sulfonamides (sulfamethoxazole with trimethoprim (SFM+TMT– 1.25/23.75 µg), nitrofurans (furadonin [FD] – 300 µg, and furazolidone (FZ)– 300 µg). The data obtained were interpreted in accordance with EUCAST 2021 (EUCAST, 2021) and Clinical and Laboratory Standards Institute (CLSI) 2019 (CLSI, 2019). Isolates of bacteria that are resistant to at least three classes of antibiotics have been classified as multidrug-resistant microorganisms [24].

To detect the genes of resistance to antibacterial agents, the DNA of bacterial samples demonstrating a similar property was examined by PCR using the following primers: for the group of beta-lactam penicillins (*bla*TEM, *bla*SHV, and OXA), aminoglycosides (*aph*A1 and *aad*B), tetracyclines (*tet*A and *tet*B), quinolones (*qnr*A and *qep*A), and sulfonamides (*sul*3) (Table-3) [24–27].

**Table-3:** Primers used for antibiotic resistance genes and virulence detection.

Primer	Sequence (5’- 3’)	Amplicon size	Reference
*bla*TEM	GAGTATTCAACATTTTCGT ACCAATGCTTAATCAGTGAG	608	[24]
*sul*1	TTCGGCATTCTGAATCTCAC ATGATCTAACCCTCGGTCTC	436	[24]
*sul*3	GAGCAAGATTTTTGGAATCG CATCTGCAGCTAACCTAGGGCTTTGA	880	[24]
*qnr*A	ATTTCTCACGCCAGGATTTG GATCGGCAAAGGTTAGGTCA	516	[24]
*qep*A	GCAGGTCCAGCAGCGGGTAG CTTCCTGCCCGAGTATCGTG	218	[24]
*aph*A1	AAACGTCTTGCTCGAGGC CAAACCGTTATTCATTCGTGA	500	[24]
*aad*B	ATGGACACAACGCAGGTCGC TTAGGCCGCATATCGCGACC	634	[24]
*tet*A	GCTACATCCTGCTTGCCT CATAGATCGCCGTGAAGA	210	[25]
*tet*B	CATTAATAGGCGCATCGCTG TGAAGGTCATCGATAGCAGG	930	[26]
*OX*A	TCAACAAATCGCCAGAGAAG TCCCACACCAGAAAAACCAG	276	[27]

The reaction mixture consisted of DreamTaq Green Master Mix (Thermo Fisher Scientific Inc., USA), water, 10 pmol1 primers, and the study DNA. As shown in Table-4, the amplification mode was selected for each pair of primer. A 100 bp marker, Tris-borate-EDTA buffer solution, and SYBR Safe DNA gel stain dye (Thermo Fisher Scientific Inc.) were used to set the reaction conditions.

**Table-4:** Cycling conditions of the primers during PCR.

Gene	Primary denaturation	Secondary denaturation	Annealing	Extension	No. of cycles	Final extension
*bla*TEM	95°C 5 min	95°C 45 s	55°C 45 s	72°C 45 s	30	72°C 10 min
tetA	95°C 5 min	95°C 30 s	60°C 1 min	72°C 1 min	30	72°C 10 min
*tet*B	94°C 5 min	94°C 40 s	64°C 45 s	72°C 45 s	32	72°C 5 min
*OX*AI	94°C 2 min	94°C 20 s	48°C 30 s	72°C 30 s	30	72°C 3 min
*sul*1	94°C 5 min	94°C 40 s	64°C 45 s	72°C 45 s	30	72°C 5 min
sul3	94°C 5 min	94°C 1 min	53°C 1 min	72°C 1 min 30 s	35	72°C 10 min
*qnr*A	94°C 5 min	94°C 1 min	53°C 1 min	72°C 1 min 30 s	35	72°C 10 min
*qepA*	94°C 5 min	94°C 40 s	60°C 40 s	72°C 45 s	30	72°C 7 min
*aph*A1	94°C 5 min	94°C 40 s	60°C 40 s	72°C 45 s	30	72°C 7 min
*aad*B	95°C 5 min	94°C 45 s	57°C 45 s	72°C 1 s	30	72°C 5 s

Amplification products were detected by electrophoresis in 1.5% agarose gel (Thermo Fisher Scientific) using a Quantum ultraviolet transilluminator (1100SUPER-BRIGHT; Reqlab, Germany).

## Results

After sowing on Compact Dry EC plate, cheeses from different regions of Kazakhstan, produced both in vacuum packaging and without packaging, colonies of blue color were isolated from 93 samples (44.9%), demonstrating the morphological characteristics of *E*. *coli*. Bacterial colonies of similar colors formed due to the chromogenic substrate 5-bromo-4-chloro-3-indolyl-B-D-glucuronide. At the same time, artisan cheeses produced using artisanal methods had the highest levels of contamination.

Spectrometric analysis of the isolated microorganisms on MALDI revealed that they were identified as species of the following enterobacteria genera: *Escherichia*, *Enterobacter*, and *Citrobacter*. *E. coli* was detected more frequently (69.9%), indicating post-pasteurization contamination of dairy products from the production environment.

Subsequent bacteriological studies of 65 samples of the cheese under study in which *E. coli* was spectrometrically identified confirmed the presence and allowed the isolation of colonies of morphologically and biochemically identifiable *E. coli* bacteria.

According to the Public Health Laboratories (PHLs) hygienic standards for colony-forming unit (CFU), a large proportion of *E*. *coli*-contaminated cheese samples were satisfactory (18.4%) and acceptable (10.6%), whereas 1.9 and 0.5% of cheese samples were unsatisfactory and unacceptable, respectively. Simultaneously, the last two categories of microbiological assessment were mainly characterized by soft (6.2%) and semi-hard (0.9%) types of cheeses (Table-5).

**Table-5:** Microbiological sanitary assessment of *Escherichia coli* contaminated cheese samples, according to PHLs.

Type of cheeses	CFU/g (n, %)			

	Satisfactory	Acceptable	Unsatisfactory	Unacceptable
			
	<20	20–100	>100 CFU/g	≥ 10^4^
Soft (n=64)	18 (28.1)	9 (14)	3 (4.7)	1 (1.5)
Semi-hard (n=102)	16 (15.6)	11 (10.8)	1 (0.9)	0
Hard (n=41)	4 (28.6)	2 (4.9)	0	0
Total (n=207)	38 (18.4)	22 (10.6)	4 (1.9)	1 (0.5)

CFU=Colony-forming unit

Analysis of the geographical distribution of cheese contamination with *E*. *coli* showed that samples from the northern region had the highest percentage of detectability of these bacteria, reaching 46% of the total number of isolates, whereas the proportion of contaminated samples from the eastern and central regions was lower (29% and 25%, respectively). *E*. *coli* strains were not found in cheese samples from the southern region. In total, 31.4% of product samples were contaminated by *E*. *coli*, with soft and semi-hard cheese varieties being the most contaminated (48 and 28%, respectively), while only 15% of hard product samples were contaminated. This distribution pattern of contamination by cheese varieties was typical for all country regions.

The level of *E. coli* contamination of commercialized cheeses depended on the size of the enterprises. Therefore, approximately two-thirds of the products studied from small farms were contaminated with *E. coli* (65%), and approximately one-third of the products of large and medium-sized enterprises were found to contain *E. coli* (27.5 and 31%, respectively). Simultaneously, contamination was most often observed in all enterprises with soft cheeses, and 80% of small artisanal cheese producers were contaminated (Table-6).

**Table-6:** Dependence of the cheese contamination by *Escherichia coli* on the enterprises’ size.

Enterprises’ size	Type of cheese						Total	

	Soft		Semi-hard		Hard			
			
	Number of samples	Contamination, %	Number of samples	Contamination, %	Number of samples	Contamination, %	Number of samples	Contamination, %
Large (n = 15)	34	44.1	50	24	25	12	109	27.5
Medium (n = 7)	20	40	42	26.2	16	18.8	78	28
Small (n = 8)	10	80	10	50	0	0	20	65
	64	48.4	102	27.5	41	14.6	207	31.4

With regard to packaging methods, *E*. *coli* detection was higher in cheese samples sold by packaging at the point of sale (47.8%) than in products packed in individual vacuum packages (26.7%) under processing conditions. The highest level of contamination was noted in the soft-type cheese samples (43.1% in vacuum packaging and 100% at the point of sale). Contamination of packaged semi-hard cheese (46.2%) was lower than soft cheese but higher than hard cheese. In general, hard cheese contamination in both packaging types was lower than that in other cheese types (Table-7).

**Table-7:** Contamination of cheeses with *Escherichia coli* depending on the packaging method.

Types of cheeses	Packaging method			

	Individually vacuumed		Packaged at the sale point	
	
	Number of samples	Contamination, %	Number of samples	Contamination, %
Soft	58	43.1	6	100
Semi-hard	76	21.1	26	46.2
Hard	27	0.7	14	28.6
Total	161	26.7	46	47.8

When examining the isolates for the presence of pathogenic strains, it was found that during electrophoresis, the amplification products of the *E*. *coli* DNA primer sites of one soft cheese sample from a small farm produced unique bands of approximately 618 bp corresponding to the *eae* gene, proving the presence of the *E. coli* O157:H7 strain in the bacterial culture.

A study of *E*. *coli* strains for resistance to 20 antibacterial drugs showed that the isolates were sensitive only to drugs of the aminoglycoside group (STR, KN, GNT), 50% of the β-lactam group drugs (AMP, CFD, and CFP), and to an agent of four of the fluoroquinolones group (EFX) (Table-8).

**Table-8:** Results the antibiotic resistance testing of *Escherichia coli* strains (n = 65).

Groups of antibacterial drugs	Antibacterial drug	Interpretation criteria, number		

		Resistant (n/%)	Intermediate (n/%)	Sensitive (n/%)
*β*-lactams	AMP			65 (100)
	AMX	2 (3)		63 (97)
	CFD			65 (100)
	CFP			65 (100)
	CFN	20 (31)	2 (3)	43 (66)
	MPN	7 (11)		58 (89)
Aminoglycosides	STR			65 (100)
	KN			65 (100)
	GNT			65 (100)
Amphenicols	LEV	13 (20)		52 (80)
Tetracyclines	TET	13 (20)		52 (80)
	DOX	6 (9)		59 (91)
Quinolones	NA	2 (3)		63 (97)
Sulfonamides	TMT+SFM	8 (12)		57 (88)
Fluoroquinolones	CFX	2 (3)		63 (97)
	NFX	3 (5)		62 (95)
	EFX			65 (100)
	OFX	4 (6)		61 (94)
Nitrofurans	FD	9 (14)	2 (3)	54 (83)
	FZ	10 (15)		55 (85)

AMP=Ampicillin, AMX=Amoxicillin, CFD=Cefpodoxime, CFP=Cefoperazone, CFN=Cefoxcitin, MPN=Meropenem, STR=Streptomycin, KN=Kanamycin, GNT=Gentamicin, LEV=Levomycetin, TET=Tetracycline, DOX=Doxycycline, NA=Nalidixic acid, TMT+SFM=Trimethoprim/sulfamethoxazole, CFX=Ciprofloxacin, NFX=Norfloxacin, EFX=Enrofloxacin, OFX=Ofloxacin, FD=Furadonin, FZ=Furazolidone

It should be noted that 1.5%, 3.1%, 4.6%, 6.2%, and 20% of isolates were simultaneously resistant to eight antibacterial drugs, seven, six, five, four, three, two, and one drug, respectively (Table-9).

**Table-9:** Profile of resistance to antibacterial agents of *Escherichia coli* isolates (n = 65).

Antibacterial drugs’ count	Antibacterial drugs’ combination	Number of resistant isolates
3	CFN, MPN, TET	2
3	CFN, LEV, TMT+SFM	2
4	CFN, LEV, FD, FZ	2
4	CFN, LEV, DOX, FZ	2
5	CFN, MPN, LEV, TET, FD	2
5	CFN, MPN, LEV, TET, DOX	1
6	CFN, MPN, LEV, TET, DOX, FD	1
6	CFN, LEV, TET, NFX, NA, FD	1
7	CFN, LEV, TET, FD, NFX, NA, FZ	1
7	CFN, AMX, DOX, FD, NFX, TMT+SFM, FZ	1
8	CFN, CFX, AMX, MPN, LEV, TET, DOX, FD	1

AMX=Amoxicillin, CFN=Cefoxcitin, MPN=Meropenem, LEV=Levomycetin, TET=Tetracycline, DOX=Doxycycline, NA=Nalidixic acid, TMT+SFM=Trimethoprim/sulfamethoxazole, CFX=Ciprofloxacin, NFX=Norfloxacin, FD=Furadonin, FZ=Furazolidone

A molecular genetic study of the presence of resistance to antibacterial drug genotypes among isolated *E*. *coli* isolates identified genes encoding resistance to β-lactams in 15.4%, sulfonamides in 30.8%, and quinolones in 9.3% (Table-10).

**Table-10:** Genes for antibiotic resistance in cheese samples (n = 65) of Kazakhstani producers.

Groups of antibacterial drugs	Antibiotic resistance genes	Number of positive samples	Proportion, %
β-lactams	*blaTEM*	10	15.4
Aminoglycosides	*aadA, aphA1*	0	0
Tetracyclines	*tetA tetB*	0	0
Sulfonamides	*sul1, sul3*	20	30.8
Quinolones	*qnrA*	6	9.3

## Discussion

This study demonstrates the relevance of microbial contamination in retail food products. Three bacterial genera belonging to the *Enterobacteriaceae* family, *Escherichia*, *Enterobacter*, and *Citrobacter*, were identified in samples of different types of cheese produced in Kazakhstan in this study. The first two genera are most often isolated from raw milk [28], and *Serratia* is also often found in commercial cheeses [29, 30]. Although coliforms are considered to be thermolabile and cannot withstand pasteurization, 44.9% of the studied cheeses were found to be contaminated with them. At the same time, approximately 70% of the contaminated samples contained *E*. *coli*, which was identified in almost a third of the samples of cheeses of various types produced in the country, and these results are comparable with those of studies of cheeses and milk in other regions of the world [31, 32].

It should be noted that, according to Kazakhstani standards, the microbiological veterinary and sanitary evaluation of dairy products is carried out only on the basis of the level of contamination with coliforms, whereas international standards suggest that such products should be examined in terms of the degree of *E. coli* contamination. Studies have shown that if assessed according to PHLs standards [33], the main proportion (92.3%) of domestic cheese contaminated with this species was acceptable, and 7.7% was unacceptable for consumption by CFU/g. At the same time, 4/5 of the products unacceptable for this indicator were soft cheeses.

The detection rate of *E. coli*-contaminated cheese samples in the northern region was relatively higher (approximately 12) than that in the central and eastern regions. This species has not been found in cheeses from the southern region, probably due to the small number of samples studied in the region. In all geographical locations where *E. coli* was detected in the products, soft cheeses without vacuum packaging (100%) were the most contaminated. In addition, contamination of cheeses with bacteria was also correlated with the size of the enterprise. Therefore, the level of product contamination of small farms was more than twice that of large and medium-sized producers, and *E. coli* was isolated in 65% of the cheese samples sold.

At present, there is an increase in the global demand for farm cheeses, as consumers increasingly prefer distinctive products with regional flavor, characteristic tastes, and characteristics [34]. As a means of income diversification, many farmers prefer manual small-scale handicraft production, and this cheese-making is becoming popular among the population of remote mountainous and steppe regions of Central Asia. However, as has been shown in the present studies, such production does not appear to comply with proper hygiene requirements, leading to high contamination of the final product with coliforms and other microflora. In some European countries, in particular France, up to 25.0% of samples of farm cheeses were positive for pathogenic STEC [35].

It should be noted that the pathogenic *E. coli* O157:H7 strain was detected for the first time in Central Asia from a sample of soft cheese from a small-scale producer in this study. The level of contamination of domestic cheese samples with this strain was 0.5%. In the past two decades of this century, endemic outbreaks of *E. coli* O157:H7 toxic infection among humans caused by cheese consumption have been documented in France [36], the USA [37], and Canada [38]. Therefore, food contamination by STEC is the greatest concern to public health in most countries, and the Food Safety System (FSS)/Food Standard Agency (FSA) considers any STEC strain as potentially pathogenic [39]. In all cases of *E. coli* O157:H7 outbreaks caused by cheese consumption, improper hygienic production conditions, such as lack of effective cleaning and disinfection procedures, lack of sanitary treatment, and insufficient cheese aging time, have been identified in enterprises where contaminated products were produced. Microorganisms can grow in biofilms in dairy production equipment, creating ideal conditions for the long-term survival of *E. coli* in the environment, which threatens the food safety of commercial products [40].

According to recent epidemiological studies, EPEC averaged 5.0% in the etiological structure of acute diarrhea in children in Kazakhstan; only serotypes 020, 0111, and 055 of this species have been described in the country [41]. The first detection of *E*. *coli* O157:H7 in cheese suggests the possibility of contamination of dairy products with other STEC strains. To fully monitor the food safety of animal products sold, thoroughly studying the microbial landscape in relation to this *E. coli* pathogenic group is necessary.

The presence of antimicrobial-resistant bacteria in dairy products is recognized as a global problem of food safety and public health due to the potential transmission risk of resistant pathogens to the human population [18, 19]. The resistance of *E*. *coli* strains isolated from cheeses produced in Kazakhstan to antibacterial agents showed that bacterial isolates were resistant to 65% of the 20 drugs studied. The proportions of AMX, NA, and CFX-resistant isolates were 3%, 5%, 6%, 9%, 11%, 11%, 12%, 12%, 14%, 15%, 20%, and 31% for NFX, OFX, DOX, MPN, SFM+TMT, FD, FZ, LEV, TET, 20 and CFN, respectively. The data obtained are consistent with the results of many studies conducted in different countries, where it was found that resistance to sulfonamides, tetracyclines, and aminoglycosides, in general, is the most common among *E. coli* strains isolated from food [42]. It should be noted that the resistance of 16 isolated *E. coli* isolates (25% of the total number studied) was multi-resistance because they were resistant to three or more classes of antimicrobial drugs. Similar results have been reported in many previous studies, where *E. coli* strains with multidrug resistance have been detected in food and environment [13, 43–45].

Thus, *E. coli* strains resistant to antimicrobial drugs are widespread in the production environment of dairy enterprises in Kazakhstan and can seriously threaten public health because such bacteria can carry and transmit genes encoding determinants of antibiotic resistance [46]. Molecular studies of *E. coli* isolates in the framework of this work confirmed the presence of *eaeA* virulence and resistance genes to β-lactam, sulfonamide, and quinolone groups. These genes are often associated with mobile genetic elements, such as plasmids, bacteriophages, and transposons, which can be exchanged between bacteria belonging to different strains and phylogenetic lines and create new combinations of virulence and resistance factors [43]. As a result, virulent and antibacterial-resistant bacteria acting as a reservoir can transmit these genes to commensal and pathogenic microorganisms inside the human digestive tract [45] and increase the incidence of foodborne diseases [39, 47, 48].

Thus, the results of these studies indicate that in Kazakhstan, there are potential risks for increasing the antibiotic resistance of the intestinal microbiome, as well as endemic outbreaks of food infections and intoxication of the human population associated with contamination by *E. coli* in cheeses produced in the country. Therefore, to supplement the regulatory documents of Kazakhstan on monitoring and control of food coliform enteropathogens, we consider it relevant to further comprehensive research on domestic and imported dairy products’ contamination with *E. coli*. This investigation will be of great scientific and practical importance for dairy businesses and veterinary and medical services in the context of the One Health concept and to ensure food safety in the country.

## Conclusion

*E. coli* contamination was found in 31.4% of commercial Kazakh cheese samples of different brands, regardless of the geographical location and capacity of dairy enterprises. According to microbiological indicators, soft cheeses (65% of samples) from small farmers (80%) packaged at retail locations (100%) were the most contaminated with *E. coli*. For the first time in Central Asia, the enteropathogenic strain *E. coli* O157:H7 was detected in 0.5% of cheese samples. *E. coli* isolates from cheese samples were resistant to 65% of the tested antibacterial drugs and contained resistance genes to β-lactams, sulfonamides, and quinolones groups. These results confirm the need to develop effective mechanisms to control the production and sale of ready-to-eat dairy products in the event of contamination by *E*. *coli* and its pathogenic and multi-resistant antibacterial drug strains.

## Authors’ Contributions

AK: Collected the samples, conducted experiments, and drafted the manuscript. AU and AA: Analyzed the experimental data, designed the study, and drafted the manuscript. ZA: Performed the microbiological study of samples. RR: Performed the genotypic research. DS: Performed antibacterial resistance studies. LS: Performed the mass-spectrometric research. SR and EA: Performed the data analysis. All authors have read, reviewed, and approved the final manuscript.

## References

[1] Hassan G.M, Gomaa S.M (2016). Microbiological quality of soft cheese marketed in Cairo and Giza governorates. Alex. J. Vet. Sci.

[2] Waldman K.B, Kerr J.M (2018). Does safety information influence consumers'preferences for controversial food products?. Food Qual. Prefer.

[3] Donnelly C (2018). Review of Controls for Pathogen Risks in Scottish Artisan Cheeses Made from Unpasteurised Milk. Vol. 4. Food Standards Scotland, Scotland.

[4] Hammad A.M, Eltahan A, Hassan H.A, Abbas N.H, Hussien H, Shimamoto T (2022). Loads of coliforms and fecal coliforms and characterization of thermotolerant *Escherichia coli* in fresh raw milk cheese. Foods.

[5] Bagel A, Sergentet D (2022). Shiga toxin-producing *Escherichia coli* and milk fat globules. Microorganisms.

[6] Heiman K.E, Garalde V.B, Gronostaj M, Jackson K.A, Beam S, Joseph L, Silk B.J (2016). Multistate outbreak of listeriosis caused by imported cheese and evidence of cross-contamination of other cheeses, USA, 2012. Epidemiol. Infect.

[7] Sauders B.D, D'Amico D.J (2016). *Listeria monocytogenes* cross-contamination of cheese:Risk throughout the food supply chain. Epidemiol. Infect.

[8] Jang J, Hur H.G, Sadowsky M.J, Byappanahalli M.N, Yan T, Ishii S (2017). Environmental *Escherichia coli*:Ecology and public health implications-a review. J. Appl. Microbiol.

[9] Gaulin C, Levac E, Ramsay D, Dion R, Ismail J, Gingras S, Lacroix C (2012). *Escherichia coli* O157:H_7_ outbreak linked to raw milk cheese in Quebec, Canada:Use of exact probability calculation and case study approaches to foodborne outbreak investigation. J. Food Prot.

[10] Currie A, Galanis E, Chacon P.A, Murray R, Wilcott L, Kirkby P, Flint J (2018). Outbreak of *Escherichia coli* O157:H_7_ infections linked to aged raw milk gouda cheese, Canada, 2013. J. Food Prot.

[11] Sodagari H.R, Wang P, Robertson I, Abraham S, Sahibzada S, Habib I (2021). Antimicrobial resistance and genomic characterisation of *Escherichia coli* isolated from caged and non-caged retail table eggs in Western Australia. Int. J. Food Microbiol.

[12] Jamali H, Paydar M, Radmehr B, Ismail S, Dadrasnia A (2015). Prevalence and antimicrobial resistance of *Staphylococcus aureus* isolated from raw milk and dairy products. Food Control.

[13] Bendary M.M, Abdel-Hamid M.I, Alshareef W.A, Alshareef H.M, Mosbah R.A, Omar N.N, Moustafa W.H (2022). Comparative analysis of human and animal *E. coli:* Serotyping, antimicrobial resistance, and virulence gene profiling. Antibiotics (Basel).

[14] Wolfe B.E, Button J.E, Santarelli M, Dutton R.J (2014). Cheese rind communities provide tractable systems for *in situ* and *in vitro* studies of microbial diversity. Cell.

[15] Marozzi S, De Santis P, Lovari S, Condoleo R, Bilei S, Marcianò R, Mezher Z (2016). Prevalence and molecular characterisation of Shiga toxin-producing *Escherichia coli* in raw milk cheeses from Lazio region, Italy. Ital. J. Food Saf.

[16] Farrokh C, Jordan K, Auvray F, Glass K, Oppegaard H, Raynaud S, Cerf O (2013). Review of Shiga-toxin-producing *Escherichia coli* (STEC) and their significance in dairy production. Int. J. Food Microbiol.

[17] Jaakkonen A, Salmenlinna S, Rimhanen-Finne R, Lundström H, Heinikainen S, Hakkinen M, Hallanvuo S (2017). Severe outbreak of sorbitol-fermenting *Escherichia coli* O157 via unpasteurized milk and farm visits, Finland 2012. Zoonoses Public Health.

[18] Abdel-Rahman M.A.A, Hamed E.A, Abdelaty M.F, Sorour H.K, Badr H, Hassan W.M, Shalaby A.G, Halem A.A.E, Soliman M.A, Roshdy H (2023). Distribution pattern of antibiotic resistance genes in *Escherichia coli* isolated from colibacillosis cases in broiler farms of Egypt. Vet. World.

[19] Putra A.R, Effendi M.H, Koesdarto S, Suwarno S, Tyasningsih W, Estoepangestie A.T (2020). Detection of the extended spectrum β-lactamase produced by *Escherichia coli* from dairy cows by using the Vitek-2 method in Tulungagung regency, Indonesia. Iraqi J. Vet. Sci.

[20] Dell'Orco F, Gusmara C, Loiacono M, Gugliotta T, Albonico F, Mortarino M, Zecconi A (2019). Evaluation of virulence factors profiles and antimicrobials resistance of *Escherichia coli* isolated from bulk tank milk and raw milk filters. Res. Vet. Sci.

[21] Federal Agency on Technical Regulating and Metrology. Food-Stuffs Methods for Detection and Determination of *Escherichia coli.* GOST 30726-2001.

[22] International Organization for Standardization (2001). Microbiology of Food and Animal Feeding Stuffs-Horizontal Method for the Enumeration of Beta-Glucuronidase-Positive *Escherichia coli*-Part 2:Colony-Count Technique at 44 Degrees C Using 5-Bromo-4-Chloro-3-Indolyl Beta-D-Glucuronide (ISO 16649-2:2001) International Organization for Standardization, Switzerland.

[23] Verocytotoxigenic *Escherichia coli* (2018). OIE Terrestrial Manual.

[24] El Kojok H, Khalil M, Hage R, Jammoul R, Jammoul A, El Darra N (2022). Microbiological and chemical evaluation of dairy products commercialized in the Lebanese market. Vet. World.

[25] Rychshanova R, Ruzauskas M, Chuzhebayeva G, Mockeliunas R, Mamiyev N, Virgailis M, Shevchenko P, Siugzdiniene R, Anskiene L, Mendybayeva A (2021). Differences in antimicrobial resistance of *Salmonella spp*. Isolated from humans, animals and food products in Kazakhstan. J. Hell. Vet. Med. Soc.

[26] Memon J, Kashif J, Hussain N, Yaqoob M, Ali A, Buriro R, Hongjie F (2016). Serotypes, genotypes, virulence factors and antimicrobial resistance genes of *Escherichia coli* isolated in bovine clinical mastitis from Eastern China. Pak. Vet. J.

[27] Abdel-Rahim M.H, El-Badawy O, Hadiya S, Daef E.A, Suh S.J, Boothe D.M, Aly S.A (2019). Patterns of fluoroquinolone resistance in *Enterobacteriaceae* isolated from the Assiut University Hospitals, Egypt:A comparative study. Microb. Drug Resist.

[28] Parussolo L, Sfaciotte R.A.P, Dalmina K.A, Melo F.D, Da Costa U.M, Ferraz S. M (2019). Detection of virulence genes and antimicrobial resistance profiles of *Escherichia coli* isolates from raw milk and artisanal cheese in Southern Brazil. Semina Ciências Agrárias.

[29] Masiello S.N, Martin N.H, Trmčić A, Wiedmann M, Boor K.J (2016). Identification and characterization of psychrotolerant coliform bacteria isolated from pasteurized fluid milk. J. Dairy Sci.

[30] Jackson E.E, Erten E.S, Maddi N, Graham T.E, Larkin J.W, Blodgett R.J, Reddy R.M (2012). Detection and enumeration of four foodborne pathogens in raw commingled silo milk in the United States. J. Food Prot.

[31] Travert B, Rafat C, Mariani P, Cointe A, Dossier A, Coppo P, Joseph A (2021). Shiga toxin-associated hemolytic uremic syndrome:Specificities of adult patients and implications for critical care management. Toxins (Basel).

[32] Elbehiry A, Marzouk E, Moussa I.M, Alenzi A, Al-Maary K.S, Mubarak A.S, Attala O.A (2021). Multidrug-resistant *Escherichia coli* in raw milk:Molecular characterization and the potential impact of camel's urine as an antibacterial agent. Saudi J. Biol. Sci.

[33] Irlinger F, Layec S, Hélinck S, Dugat-Bony E (2015). Cheese rind microbial communities:Diversity, composition and origin. FEMS Microbiol. Lett.

[34] Gilbert R.J, De Louvois J, Donovan T, Little C, Nye K, Ribeiro C.D, Bolton F.J (2000). Guidelines for the microbiological quality of some ready-to-eat foods sampled at the point of sale. PHLs advisory committee for food and dairy products. Commun. Dis. Public Health.

[35] Donnelly C.W (2013). From Pasteur to probiotics:A historical overview of cheese and microbes. Microbiol. Spectr.

[36] Vernozy-Rozand C, Mazuy-Cruchaudet C, Bavai C, Montet M.P, Bonin V, Dernburg A, Richard Y (2005). Growth and survival of *Escherichia coli* O157:H_7_ during the manufacture and ripening of raw goat milk lactic cheeses. Int. J. Food Microbiol.

[37] Espie E, Vaillant V, Mariani-Kurkdjian P, Grimont F, Martin-Schaller R, De Valk H, Vernozy-Rozand C (2006). *Escherichia coli* O157 outbreak associated with fresh unpasteurized goats'cheese. Epidemiol. Infect.

[38] Honish L, Predy G, Hislop N, Chui L, Kowalewska-Grochowska K, Trottier L, Zazulak I (2005). An outbreak of *E. coli* O157:H_7_ hemorrhagic colitis associated with unpasteurized gouda cheese. Can. J. Public Health.

[39] Awosile B, Eisnor J, Saab M.E, Heider L, McClure J.T (2021). Occurrence of extended-spectrum β-lactamase and AmpC-producing *Escherichia coli* in retail meat products from the Maritime Provinces, Canada. Can. J. Microbiol.

[40] EFSA Biohaz Panel Koutsoumanis K, Allende A, Alvarez-Ordóñez A, Bover-Cid S, Chemaly M, Bolton D (2020). Pathogenicity assessment of Shiga toxin-producing *Escherichia coli* (STEC) and the public health risk posed by contamination of food with STEC. EFSA J.

[41] Marchand S, De Block J, De Jonghe V, Coorevits A, Heyndrickx M, Herman L (2012). Biofilm formation in milk production and processing environments;Influence on milk quality and safety. Compr. Rev. Food Sci. Food Saf.

[42] Baymuratova M.A, Bazarova G.S, T'yesova-Berdalina R.A, Zhumabekova B.T, Abdusalamova Z.S, Aneshova E.Y (2018). The efficacy of children intestinal escherichiosis microbiological monitoring in Almaty [Effektivnost'mikrobiologicheskogo monitoringa za kishechnymi esherikhiozami u detey g. Almaty]. Vestnik AGIUV.

[43] Islam M.S, Nayeem M.M.H, Sobur M.A, Ievy S, Islam M.A, Rahman S, Rahman M.T (2021). Virulence determinants and multidrug resistance of *Escherichia coli* isolated from migratory birds. Antibiotics (Basel).

[44] Ammar A.M, Abd El-Hamid M.I, El-Malt R.M, Azab D.S, Albogami S, Al-Sanea M.M, Bendary M.M (2021). Molecular detection of fluoroquinolone resistance among multidrug-, extensively drug-, and pan-drug-resistant *Campylobacter* species in Egypt. Antibiotics (Basel).

[45] Wellington E.M, Boxall A.B, Cross P, Feil E.J, Gaze W.H, Hawkey P.M, Williams A.P (2013). The role of the natural environment in the emergence of antibiotic resistance in gram-negative bacteria. Lancet Infect. Dis.

[46] Othman S.M, Sheet O.H, Al-Sanjary R (2023). Phenotypic and genotypic characterizations of *Escherichia coli* isolated from veal meats and butchers'shops in Mosul city, Iraq. Iraq J. Vet. Sci.

[47] Jafari-Sales A, Hosein-Nezhad P, Shahniani A (2020). Antibiotic susceptibility assessment of *Escherichia coli* isolated from traditional cheeses in Marand, Iran. Int. J. Adv. Biol. Biomed. Res.

[48] Condoleo R, Palumbo R, Mezher Z, Bucchini L, Taylor R.A (2022). Microbial risk assessment of *Escherichia coli* Shiga-toxin producers (STEC) in raw sheep's milk cheeses in Italy. Food Control.

